# Effective Parameters on Fabrication and Modification of Braid Hollow Fiber Membranes: A Review

**DOI:** 10.3390/membranes11110884

**Published:** 2021-11-17

**Authors:** Azadeh Nazif, Hamed Karkhanechi, Ehsan Saljoughi, Seyed Mahmoud Mousavi, Hideto Matsuyama

**Affiliations:** 1 Department of Chemical Engineering, Faculty of Engineering, Ferdowsi University of Mashhad, Mashhad 9177948974, Iran; azadehnazif@gmail.com (A.N.); saljoughi@um.ac.ir (E.S.); mmousavi@um.ac.ir (S.M.M.); 2 Research Center for Membrane and Film Technology, Department of Chemical Science and Engineering, Kobe University, 1-1 Rokkodai, Nada-ku, Kobe 657-8501, Japan

**Keywords:** braid hollow fiber membrane, fabrication parameters, mechanical strength, braid reinforcing

## Abstract

Hollow fiber membranes (HFMs) possess desired properties such as high surface area, desirable filtration efficiency, high packing density relative to other configurations. Nevertheless, they are often possible to break or damage during the high-pressure cleaning and aeration process. Recently, using the braid reinforcing as support is recommended to improve the mechanical strength of HFMs. The braid hollow fiber membrane (BHFM) is capable apply under higher pressure conditions. This review investigates the fabrication parameters and the methods for the improvement of BHFM performance.

## 1. Introduction

Membrane technology, including polymeric membranes, is one of the best-advanced separation and treatment systems that have been widely used in different applications such as desalination, wastewater treatment, oil/water separation, and water reuse applications [1,2]. Hollow fiber membranes (HFMs) possess desired and competitive advantages relative to flat-sheet membranes for many membrane separation applications due to high membrane surface area per volume of a module (e.g., the ratio of area per volume is reported 40 m^2^/m^3^ for flat sheet and 170 m^2^/m^3^ for HFMs [3]). They also have high permeability and porosity, desirable filtration efficiency, proper mechanical properties, self-supported structure and characteristics, small footprint, high packing density relative to other configurations, ease of handling and maintenance [4,5,6,7,8,9,10,11,12]. Due also to the spacer-free module, the assembly cost will be reduced. Hollow fiber membranes had been widely used for microfiltration and ultrafiltration alone or as the pretreatment of nanofiltration and reverse osmosis in seawater desalination, forward osmosis, and membrane bioreactor to the treatment of industrial wastewater (such as medicine, food, and textiles) and generation of drinking water. Hollow fiber membranes are often possible to break or damage during high-pressure cleaning, module preparation, aeration process. It is due to sponge-like and asymmetric finger-like morphology that led to making brittle and porous structures. Hence, their lifetime may reduce despite their many advantages [4,11,13].

In order to design membrane structure, lifetime prediction and reliability, understanding, and analysis is important to evaluate the mechanical behavior under actual operating conditions. Mechanical abrasion of membranes arising from physical and chemical damage by harsh feed water, fouling, chemical cleaning, and back-washing bring about the reduction in membrane strength [4,14]. Generally, the mechanical properties of polymeric flat sheet membranes are improved by a polyester support layer. The nonwoven polyester possesses strong mechanical strength can tolerate vigorous hydraulic impact [15,16,17,18,19]. Hosseini et al. [15] utilized polyester support in order to improve the mechanical strength in high pressure. Recently, using tubular braid (or threads/fabric) as reinforced support is proposed to improve the mechanical strength of hollow fiber membranes. Braid-reinforced hollow fiber membranes have attracted attention and interest due to their low cost, efficient separation, relatively simple preparation, and high mechanical properties [6,7,11,20,21]. This type of hollow fiber membrane possesses a supreme tensile strength (contributes to the long lifetime of the membranes), and thus they could apply under higher pressure conditions relative to common hollow fiber membranes. A braid hollow fiber membrane (BHFM) is fabricated by coating a thin film on the surface of tubular braid (i.e., reinforced fiber). The presence of braid support increases the flux of ultrafiltration/microfiltration due to the thinner thickness of the selective layer, thanks to tolerating relatively higher pressure compared with the typical HFM [4,21,22,23,24]. The first studies in reinforced HFMs are related to the patents. Cooper et al. [25] introduced the concept of braided membrane for the first time. They cast the membrane on a supporting surface such as fabric-like material consisting of monofilament material (e.g., polyesters, nylon, rayon, polyolefin, Teflon, acrylic) with a small diameter. Zenon Environmental Inc. produced a type of hollow fiber membrane consisting of tubular macro-porous support and a tubular semipermeable thin film of the polymer. The prepared braid hollow fiber membrane could endure to 10.3 MPa in hydraulic compaction forces [26].

The peeling of the surface layer from the tubular braid is the drawback of the braid hollow fiber membranes due to thermodynamic incompatibility between these two layers [21]. The braid hollow fiber membrane can significantly enhance the effective area due to fewer sticking fibers together in the assembled module [27]. The braid support absorbs the molecules of water due to the porous structure. The thin separation layer of the BHFM also contributed to the water flux enhancement because of lower thickness compared to self-support HFMs [24]. Chen et al. [28] reported that the flux of braid PMIA- BHFMs was higher than the PMIA-HFMs. It is due to the PMIA-BHFMs containing an inner layer with a relatively porous structure that leads to a reduction in membrane resistance for water transfer. The BHFMs with the dense outer surface can prevent the adsorption of foulants and the pore-blockage in the inner pores of the membrane. Therefore, the occurred fouling is forming the cake layer type, can easily remove by washing, while the open pores of the HFM easily adsorbed the molecules of protein. In this case, the pores of the membrane will be blocked. This type of fouling is irreversible and hardly eliminated through water washing. Therefore, the case of irreversible fouling required a combination of chemical cleaning and back-washing [29].

**Figure 1:**
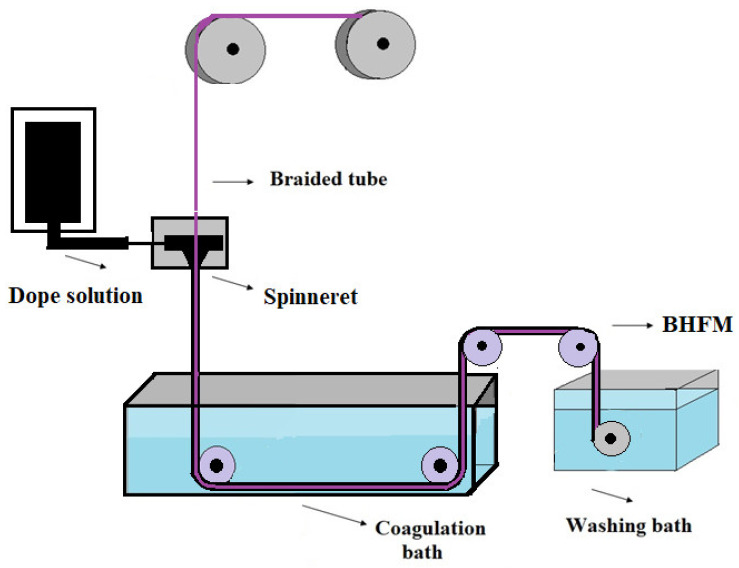
Schematic illustration of the fabrication of braid-reinforced hollow fiber membranes (BHFM).

BHFMs are fabricated by two spinning methods: electrospinning method and non–solvent-induced phase inversion (NIPS) based on the dry-wet spinning. Based on the literature, membranes prepared by the NIPS method based on the dry-wet spinning process are more common and have higher water flux due to thin separation layers [24]. As shown in Figure 1, the tube braid (like the bore fluid injection in the fabrication of common HFMs) is inserted through the middle of the spinneret. Then the polymer solution is uniformly coated on the braid tube. The prepared braid hollow fiber membrane is immersed into a coagulation bath, and it is finally wound up on the drum [4,30].

**Figure 2:**
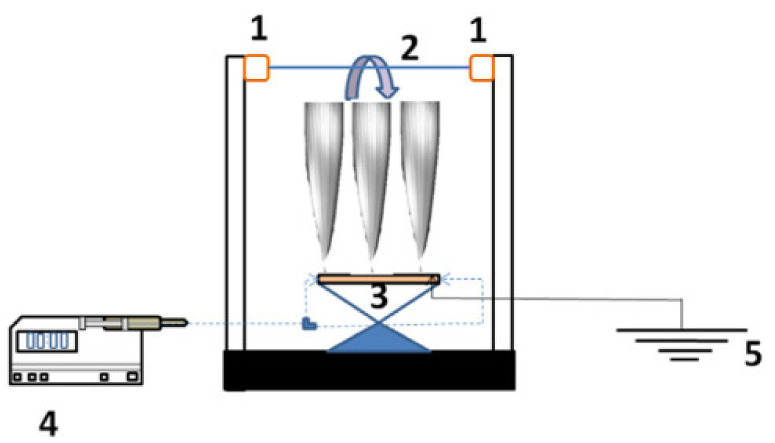
Schematic diagram of an electrospinning setup [31].

Recently, the electrospinning method has been attracted much attention to generating polymer fibers in the range of several microns to nanometer diameter (50 nm and 10 µm). Desirable properties (functionality, porosity, weight, and strength) can be achieved by the type of polymer and efficient control of operating conditions. A large specific area, a high ratio of length to diameter, and uniform pore size distribution can be achieved by the electrospinning method. The base of this method is a high-voltage electric field for the production of nanofibers from a polymeric stream that is released by a nozzle system. This technique contributes to producing of ultrathin layers from different fibers, particles, and polymers. The generated fibers from this method are affected by polymeric solution properties (concentration and the molecular weight of polymer), environmental conditions (humidity and room temperature), and operation parameters (solution flow rate, applied voltage, and tip-collector distance). In the electrospinning method, the syringe fills with a polymeric solution, then pumps to the nozzle at a specified flow rate. The braid layer is located on a thin cylindrical that is joined to the rotating shaft. The fibers will be generated by coating the polymer solution on the braid layer by applying the electric power to the nozzle (Figure 2) [31,32]. Aslan et al. [31] fabricated tubular electrospun nanofiber membranes as microfiltration membranes. Poly acrylonitrile (PAN) nanofibers coated on the braided rope. The morphology and filtration characterization showed excellent properties in terms of cross-section thickness, water flux, turbidity, porosity, hydrophilicity, and uniform distribution of pore size.

**Figure 3:**
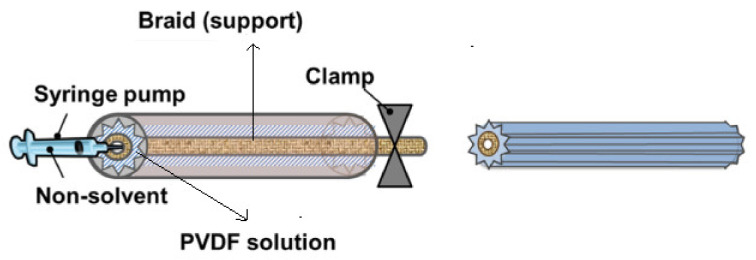
The schematic of patterned BHFM (b) low injection rate of non-solvent (c) high injection rate of non-solvent [5].

Kim et al. [5] introduced patterned morphology (prism and pyramid) to the surface of BHFM, as shown in Figure 3, by aiming to decline the fouling in MBR for wastewater treatment. The injection rate of the non–solvent mainly affected the morphology of the membranes. Uniform distribution of macro voids observed for the high injection rate on the total cross-section surface. In a low injection rate, a dense and thick polymer film was formed inward and outward of the braid, and large macro-voids were created external side of the polymer film in the vicinity of the braid. This observation can be explained based on the infiltration of the non-solvent. In a low injection rate, the non-solvent would induce phase inversion inside and near the braid relative to infiltration further to the braid. Hence, the bulk of the present polymer outside the braid diffuses toward the braid that leading to coagulation. Therefore, a dense and thick polymer film was formed inside or near the braid, and large macro voids were created outside the braid because of an insufficient content of the polymer. In a high injection rate, the non-solvent diffuses quickly in the total area of the polymeric solution. Hence, phase inversion would happen with more speed all over the polymer solution relative to the migration of the polymer to the braid. Therefore, a more uniform distribution of macro voids is created.

There is a limited amount of papers that reviewed the BHFMs [33,34]. We specially reviewed the effective fabrication parameters, modification, and performance of braid hollow fiber membranes in the different applications. Hence, the type and content of polymer, additive, the methods for increasing interfacial bonding between the braid and separation layer, and other efficient parameters are discussed.

## 2. Effective Fabrication Parameters

Dope and braid composition such as type and concentration of polymer and additive, fabrication parameters, and operational conditions play vital roles in the success of the BHFM for different applications. Accordingly, the researchers investigated the various aspects to enhance the performance of BHFMs for different applications. 

### 2.1. Type of Polymer in a Dope Solution

The selection of material is an essential factor in the achievement of desired performance in membrane application. Polymeric membranes are the most used membranes for different applications with high design flexibility [35]. Several polymers have been employed for the preparation of BHFM, such as polyacrylonitrile (PAN), poly (vinyl chloride) (PVC), cellulose acetate (CA), polysulfone (PSf), and polyvinylidene difluoride (PVDF). Table 1 provides the properties of polymers used for the preparation of BHFM. Table 1 present the used polymers for BHFM and their properties.

PVC is one of the usual and promising polymers for membrane application. However, fouling problems can restrict the PVC membranes in water application owing to their hydrophobic nature. The blending of an amphiphilic copolymer and PVC in the dope solution is one of the methods that can overcome the antifouling properties. The hydrophilic part of the amphiphilic copolymer is connected to the membrane surface, which leads to a membrane with good antifouling properties. The hydrophobic section creates good compatibility with the membrane matrix and increases the maintenance of copolymer in the membrane matrix. Zhou et al. [36] prepared the BHFM-pure PVC and blended BHFM with different blend ratios of PVC/copolymer. The bursting strength and tensile strength of BHFM were higher than 2.1 and 170 MPa, respectively, which were larger than those of the self-supporting HFMs.

Copolymers, including poly (ethylene oxide) (PEO) or poly (ethylene glycol) (PEG) chains, can create a hydration layer, preventing the binding of the foulants molecules to the surface of the membrane. However, PEG-based copolymer is suggested to improve the antifouling properties and hydrophilicity of PVC membranes. Since PEG is categorized in soft polymers, increasing the PEG content in the dope solution can reduce the mechanical strength. Hence it can limit the membrane application in practical wastewater treatment due to damaging the membrane structure during the backflush process or aeration [36]. Therefore, the optimization of PEG content is essential in order to obtain antifouling properties and desirable mechanical strength for practical application. Zhou et al. [36] used amphiphilic copolymer poly (vinyl chloride-co-poly (ethylene glycol) methyl ether methacrylate) (poly(VC-co-PEGMA)) to endow hydrophilicity to PVC braid hollow fiber membrane. Considerable improvement was observed in antifouling properties and hydrophilicity when the copolymer/PVC blending ratio in the coating solution was used in optimum content.

PAN hollow fiber membranes have been widely utilized in pervaporation, the treatment of industrial wastewater, and enzyme immobilization applications. Fabrication of the substrate for composite membranes is another PAN application due to favorable properties. Low mechanical stability limits PAN application in micro/ultrafiltration and MBR systems [7]. Quan et al. [7] prepared the PAN-BHFM by coating PAN solutions on the PET (Polyethylene terephthalate) and PAN two-dimensional braid surface. The PAN-BHFM based on the PAN braid had excellent mechanical properties due to good interfacial bonding between the polymer and the braid. It was possessed a tensile strength higher than 80 MPa.

The excellent mechanical strength and other advantages (as shown in Table 1) of PSf membrane suggest this is a good candidate for wastewater treatment systems, textile dyeing, and desalination. PSf membranes require surface modification in order to enhance hydrophilicity and water permeation [24]. The incorporation of hydrophilic nanoparticles in the PSf membrane can improve antifouling properties and increase the flux rate. Peechmani et al. [24] fabricated hybrid PSf/zinc oxide (ZnO) BHFMs to increase flux and improve hydrophilicity. The BHFMs were prepared with different concentrations of ZnO nanoparticles. The ZnO nanoparticles lead to an increase in the overall flux and absorption of water molecules onto the membrane surface due to hydrophilic nature and water-loving properties (absorption of hydroxyl groups). Increasing the hydrophilicity of the membrane surface leads to decreasing the interactions between organic matters and the membrane surface. Hence, less fouling by organic foulants happens on the hydrophilic membranes.

CA membranes are another type of polymer membrane that plays a significant role in membrane separation due to favorable properties based on Table 1. Despite the good properties of CA hollow fiber membranes, the weak mechanical strength limited their usage in practical applications such as membrane bioreactors. The HFM in the submerged MBR can easily be broken or damaged during the back-washing process or aerated airflow. Hence, it is required this type of membrane prepared with high mechanical properties. CA membrane as a hydrophilic membrane showed the high performance for antifouling properties when faced with BSA solution. The dense outer surface of BHFM could avoid or limit the blocking of the inner pore. Hence, the created fouling is mainly due to adsorption and/or deposition of pollutants on the membrane surface, which is easily eliminated by water washing [21].

One of the main aromatic polyamides is Poly (m-phenylene isophthalamide) (PMIA) with hydrophilic properties, good mechanical properties, excellent thermal stability due to the hydrogen bond network, and aramid groups. This polymer is widely used for nanofibers production and in water treatment applications. Chen et al., prepared PMIA hollow fiber membranes containing separation layers and reinforced braids. The PMIA-BHFM exhibited great antifouling property relative to PVDF membranes. The PMIA membranes exhibited a higher negativity charge relative to the PVDF membranes due to the strong polar amide groups (–NH–CO–) in the macromolecular chain of PMIA. These groups lead to creating strong electronegativity and superior hydrophilicity PMIA membranes. These properties are the main goal to improve the antifouling property [28].

### 2.2. The Effect of Polymer Concentration

**Figure 4:**
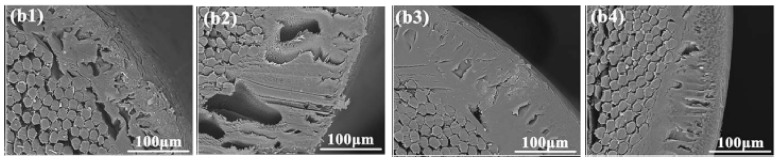
The effect of polymer concentration on BHFM structure (**b1**) 5%, (**b2**) 8%, (**b3**) 10%, and (**b4**) 15% [28].

**Figure 5:**
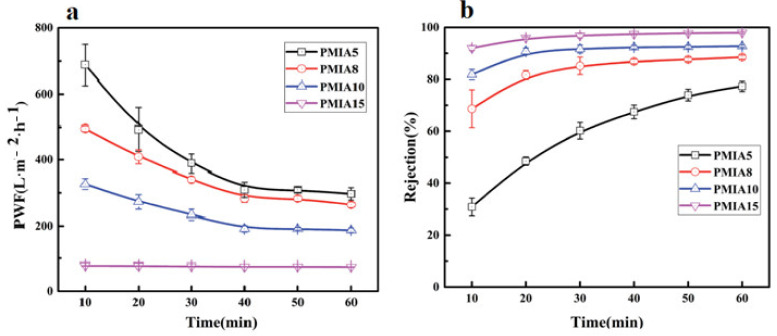
Effects of polymer concentration on the (**a**) pure water flux and (**b**) protein rejection [28].

Generally, the polymer concentration has a significant effect on membrane performance and structure. As the polymer concentration is increased in the dope solutions, the finger-like pore structure is gradually converted to a sponge-like pore structure, and the pore diameter becomes smaller. As can also be seen in Figure 4, the higher polymer concentration causes the formation of the denser and thicker skin layer. In contrast, the looser structure is reported for the membrane containing low polymer concentration. The high polymer concentration leads to increasing the viscosity of polymer solution. Therefore, the rate of diffusion is reduced between solvent and in the phase inversion process. Instantaneous demixing creates membranes with a porous layer and a finger-like structure, whereas delayed demixing results in membranes with dense structures and sponge-like pores. The dens and smooth structure and small pore size of the membrane surface cause an improvement to the antifouling ability and separation property of the membrane, though the flux will be reduced. The rejection is more dependent on the density of the separation layer compared to the structure of the cross-section [28,37,44]. Fan et al. [21] reported that the rejection of membrane with the low concentration of CA had minimum rejection due to big size pores. They also observed an increase in polymer concentration in dope solution lead to the creation of a surface with smooth and dense properties. The bursting and tensile strengths also increased due to interfacial bonding between tubular braid and separation layer. Zhang et al. [45] observed the BSA rejection for BHFM was higher than the HFM. It was due to the denser skin layer and smaller pore size in BHFM relative to HFM. The pore size is an essential factor that affects membrane permeability. As shown in Figure 5, the increase in polymer concentration in dope solution leads to decreasing of pure water flux and increasing the rejection due to lower porosity and pore size of the membrane [7,40,46].

The contact angle between the braid and the coating solution increased when the polymer concentration increased. It is due to the viscosity of polymer solution enhanced with the increase in polymer concentration; hence, the solution fluidity will be reduced [7,40,46]. 

Based on the investigation of Chen et al. [28], the antifouling properties (performance and flux recovery ratio) for PMIA BHFM were better in a higher concentration of polymer. It may be due to the presence of strong polar groups (−NH −CO) in the macromolecular chain of polymer that leads to creating excellent hydrophilicity and strong electronegativity. The other reason is the dense outer surface and small pore size in the high concentration of polymer that avoids the pore blockage of inner pores. Hence, the fouling and forming of the cake layer mainly create on the membrane surface that is easily removed in the cleaning process. 

As mentioned, the presence of the braid reinforcement leads to notable enhancement of mechanical properties. For example, Liu et al. [46] observed that the tensile strength for a membrane with three threads was more than six times relative to the membrane without thread. There is the physical and chemical force between the interface of polymer and braid layer. The physical force consists of conglutination and wedge, and chemical force created by the chemical bonds. The wettability of braid by polymer solution is the main factor that affects the interaction force between two layers. The contact angle between polymer solution and braid must be small to obtain sufficient contacting and wetting between them. With an increase in the polymer concentration, the viscosity and surface tension is increased. The burst pressure of HFM is a resisting capability in a radial direction mainly determined by force between the molecule chains of polymers. This parameter, as a critical parameter in the cleaning process, represents the interfacial bonding state of BHFM to a certain extent. In the practical application, the operating pressure is restricted by the burst pressure due to the small effect of braid thread on the radial direction persistence [21,28]. The high bursting strength would restrict the harm of BHFMS in the back-washing process. It is enhanced with the increase in polymer concentration in the dope solution due to the superior mechanical strength of the separation layer in higher polymer concentrations. There is a tighter stack of molecule chains in the membrane with higher polymer concentration. Hence, they have a higher burst pressure. There is a striking correlation between the burst pressure and operation for the normal HFM. However, adding braid threads leads to anisotropy in the transversal and longitudinal directions, so the relationship is small for BHFM. The membrane material and structure effect on burst pressure and tensile strength is influenced by braid thread. 

Fan et al. [37] observed that an increase in the polymer concentration (from 6 to 14 wt.%) in the dope solution was contributed to the enhancement of the tensile strength of CA-BHFM (from 11 to 14 MPa). Based on a study by Chen et al. [28], the mechanical strength is dominantly governed through the reinforced braids. The breaking elongation and tensile strength were approximately similar to the tensile strength of the braids. The bursting strength and initial modulus enhanced with the increase in the polymer concentration because the separation layer was firmly bonded with the braid layer that hindered the deformation of the braid layer. The separation layer also had a better mechanical strength in higher polymer concentrations. 

### 2.3. Effect of Additives

The presence of additives in bulk or on the surface membrane is one of the effective approaches to improve membrane performance through the modification of roughness, hydrophilicity, pore size, and surface charge [15]. Zhou et al. [36] fabricated the BHFM by blending PVC and different blend ratios of poly(VC-co-PEGMA) copolymer. There was a high interfacial bonding strength between the PET-braid and polymer solution. Although, the copolymers contained PEG demonstrate the strong ability of pore-forming, which leads to creating large pores and porosity enhancement. The prepared membrane exhibited antifouling resistance and high mechanical properties. The tensile strength of PVC-BHFM was significantly higher than PVC-HFM. The membrane hydrophilicity increased with increasing the copolymer concentration of the dope solution. High hydrophilicity brings about a faster demixing process and diffusion of water into the polymer solution during the membrane formation. Hence, a larger pore size will create in the selective layer compared with low hydrophilicity. The optimization of copolymer content is necessary because an increase in copolymer content based on PEG in polymer solution leads to a reduction in mechanical strength of membrane due to the PEG softness. 

Peechmani et al. [24] showed that the presence of ZnO nanoparticles in dope solution caused delay-demixing between non–solvent and solvent due to viscosity enhancement. Thus, the formation of macrovoids was consequently increased. By increasing the concentration of nanoparticles, the macrovoids and dense sponge structure and consequently higher permeation increased near the braid layer. It is due to the hydrophilic nanoparticles that contribute to the faster moving of water molecules into the membrane matrix relative to the demixing rate between non–solvent and solvent during the phase inversion process. The BSA rejection and water flux were higher contents in the highest content of ZnO compared to other membranes. It is occurred because of the hydroxyl group of ZnO nanoparticles in the selective layer that receives more water molecules. The strong electronegativity of nanoparticles also results in avoiding the deposition of BSA proteins in the selective layer. 

Lan et al. [47] fabricated a BHFM consisting of PVDF as a base polymer and PET a woven tubal as a support layer. They used TiO_2_ nanoparticles for the improvement of hydrophilicity. The prepared membranes had desirable properties in terms of the filtration area and mechanical strength relative to conventional HFM. The BHFM containing 1% TiO2 had the best antifouling property, the highest flux, and the lowest flux decline rate.

Hao et al. [38] prepared a PET-braid-reinforced PVDF hollow fiber membrane with different concentrations of graphene to increase the membrane hydrophobicity in oil-water separation application. The PET tubular braids were coated with a PVDF/graphene solution. The viscosities of polymer solutions first increased and then reduced with an increase in graphene contents. The viscosity of polymer solution varied when the shear rate increased and showed the properties of a pseudoplastic fluid. The polymer solution without graphene had a low viscosity that represented no considerable change with the enhancement of shear rate. The viscosity of the polymer solution changed clearly for the membrane with the highest amount of graphene. It is due to graphene being a laminated and rigid substance with a high Young’s modulus. In a low shear rate, the presence of tough graphene increased the flow resistance of polymer solution. With the increase in shear rate and exceeding from a specified amount, the effect of flow resistance for graphene slowly weakened, and the viscosity of the polymer solution was consequently decreased. Hao et al. observed small mean pore size and the fluctuation in thickness of the selective layer in the maximum concentration of graphene due to the high viscosity and low fluidity of polymer solution. The random distribution of graphene sheets in the selective layers of BHFMs resulted in creating a membrane with a stable pore structure due to the rigid nature of the graphene sheets. Wu et al. [43] fabricated a PU/graphene BHFM based on PET braided for oil/water separation. The prepared membranes showed good lipophilic properties based on contact angle results. Good selectivity for oil-water separation was also achieved.

Liu et al. [29] investigated the effect of molecular weights of polyethylene glycol on the structure and performance of homogenous reinforced PVC-BHFMs. The presence of PEG with high molecular weight leads to increasing the thickness of the separation layer because of the enhancement of solution viscosity. The high viscosity restricts the exchange of the solvent and non–solvent. The high molecular weight of PEG created a bigger finger-like, smooth outer surface and compact skin layer relative to the low molecular weight of PEG. The rejection of BSA protein increased, and the porosity reduced when the molecular weight of PEG was enhanced. It is due to increasing the thickness of the separation layer and forming the dense outer layer with the increase in the molecular weight of the additive. It is notable that the molecular weight of PEG did not influence the mechanical properties of prepared membranes.

### 2.4. Effect of Braid Composition 

Two reinforcing methods are reported in the literature in order to improve the improvement of mechanical properties of HFMs: fibers reinforced and porous matrix membrane reinforced as support. The fibers reinforced method is low-cost and straightforward. It could be done by the tubular braid reinforced based on the reinforcement shape and the continuous fiber-reinforced method. Lee et al. [48,49] fabricated a braid-reinforced composite HFM consisting of a tubular braid with multifilament. The multifilament is formed from monofilaments with a fineness of 0.01 to 0.4 denier. The surface area between the polymer thin film and tubular braid is increased because the fineness of the monofilaments is small. Hence, the peeling strength of the polymer thin film and tubular braid is excellent, as well as the membrane wettability is excellent because of the capillary tube phenomenon.

In the second method, the porous matrix membrane as the reinforcement is firstly prepared by melt-spinning cold-stretching or thermally induced phase separation; then, the surface coating is carried. This method is approximately high cost and complex [6]. Liu et al. [46] fabricated continuous polyester threads PVDF-HFM by incorporating PET threads in the support layer in the axial direction. They found that the tensile strength of the BHFMs improved up to 10 MPa by the increasing of PET threads number. The PET threads had low effects on the separation properties of the membrane, but the tensile strength of the membrane was increased.

Both pure and hybrid composition for the braid is reported in the literature. Fan et al. [37] prepared a homogeneous BHFM which consisted of a CA for the braid and separation layer. The prepared membrane indicated a good interfacial bonding state, but the CA fibers in the braid tend to be swollen and sick together, which decreases the permeability and flux of the membrane. Hybrid braid leads to changing porous structure, enhances the membrane separation properties, and reduces the drawback of the homogenous method. Fan et al. [21] prepared a BHFM based on a hybrid braid. The hybrid braid consists of CA and PAN. The presence of PAN fiber in hybrid composition overcame the CA fiber’s swelling and reduction in permeability. The tensile strength of prepared BHFMs increased from 16.0 MPa to 62.9 MPa by optimizing the CA/PAN ratio in the braid composition. The bursting strength was enhanced when CA fiber proportion increased in the braid. Liu et al. [6] fabricated a heterogeneous BHFM consisting of a hybrid braid (PET and PAN) and a coating layer of PVC. The prepared membrane had a desirable interfacial bonding state and tensile strength relative to the membrane containing pure PAN or PET braid. It was also observed that the tensile strength decreased when PAN filaments increased in the composition of the hybrid tubular braid.

Quan et al. [7] investigated the effect of two types of braid (PAN and PET) on membrane performance. The membrane was prepared based on PAN braid as a homogenous membrane and PET braid as a heterogeneous membrane. Their results showed that the interfacial bonding state of the PAN membrane was better than the PET membrane. The contact angle between the braid and the coating solution was lower for the PAN membrane. The tensile strength and pure water flux of the PET membrane were higher compared to the PAN membrane.

Table 2 summarizes the studies for BHFMs based on hybrid composition and pure-composition braid. The PET braid is used in most of the BHFM in the pure-composition braid. PVP (Polyvinylpyrrolidone) or PEG is generally present in dope solution as pore former. The presence of additives improves the separation property and water flux in optimum content.

### 2.5. Thin Film Composite: Braid Hollow Fiber Membranes (TFC-BHFM)

Some studies have concerned for improving properties and performance of TFC-BHFM by optimization of fabrication parameters such as soaking time, monomer ratios, reaction time, and monomer concentration in organic or aqueous solution. 

#### 2.5.1. The Effect of Monomer Concentration on TFC-BHFM

The surface properties (e.g., hydrophilicity, functional groups, crosslinking of monomers) and structure (e.g., thickness, pore dimension, and roughness) of the selective layer directly affect on the membrane performance. Thus, it is necessary the fundamental understanding of the influence of different monomers for the preparation of high-performance membranes with desirable structures [50].

**Figure 6:**
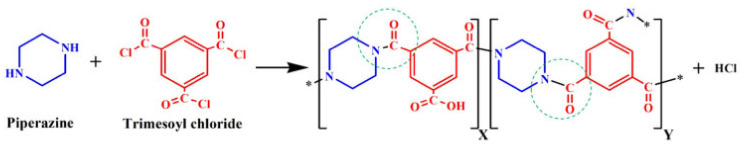
The reaction between PIP and TMC for polyamide formation [51].

Xia et al. [10] investigated the TFC-BHFMs with different monomer concentrations (PIP (piperazine) and TMC (trimesoyl chloride)). They fabricated the NF membrane that exhibited high strength at pressures up to 70 psi without fracture and created the integrity in the BHFM at pressures typically not utilized for fibers of this size by using braid-reinforced and optimization of monomer concentration. The prepared TFC-BHFM could be used for processes that salt selectivity is required. It was reported that the water permeation in the low concentration of TMC was higher in comparison with the low concentration for both monomers (i.e., PIP and TMC). It can be explained that the membrane defects are reduced, or it may be due to the increasing of the thickness or density of the selective layer in a higher monomer concentration. The different results observed from this trend in the prepared membranes with the highest content of PIP and minimum content of TMC. It is probably due to creating a thick barrier film in the lowest TMC concentration in order to stop the reaction rapidly. The high TMC concentrations had a positive effect on MgSO_4_ rejection. The different concentrations of amine groups typically cause the various permeation, which is likely due to reducing the pore size of the membrane. Figure 6 shows the reaction between TMC and PIP to form polyamide as a selective layer. 

#### 2.5.2. Effect of Soaking and Reaction Time in TFC-BHFM Preparation

The soaking of the support layer is a step of TFC-BHFM fabrication that impact significantly on the performance of prepared TFC-BHFM. Ununiformed wetting may lead to the decreasing of membrane selectivity. Using the PVDF support in the fabrication of the TFC membrane is a main challenge due to its hydrophobic nature and wetting difficulty [10,52]. Xia et al. [10] prepared the TFC-BHFMs with different soaking times. The visual properties were not changed based on the variation of soaking time. An increase in the soaking time leads to decreasing in pure water permeability, while the salt rejection firstly increases and then decreases. These results are due to forming a uniform selective layer with fewer defects after entirely wetting by the PIP solution at the longer soaking time.

The modification of support surface through plasma, coating with hydrophilic polymers (e.g., PAN and PVA), wetting of membrane by invert the sequence soaking in the organic phase and then immersion in the aqueous amine phase are the methods for wettability improvement of the of PVDF support that investigated by researchers [10]. 

Reaction time is a crucial factor in interfacial polymerization that affects the structure of the coating layer and specifies the extent of polymerization between the monomers [53]. Turken et al. [51] fabricated reinforced TFC-HF NF membranes. They selected the reinforced PSf ultrafiltration as a support and polyamide layer as a selective layer that was prepared from trimesoyl chloride (organic phases) and piperazine (aqueous phases) monomers. The immersion time of TMC was optimized in fixed concentrations of TMC and PIP to achieve the highest membrane performance. The hydrophilicity of the membrane increased by enhancement of the crosslinking degree of the polyamide layer and the TMC reaction time. Turken et al. [51] reported the higher specific permeate flux in higher TMC reaction times for reinforced-TFC-NF membranes. The hydrophilicity of the membrane enhanced when the reaction time of TMC increased. It is known that when the crosslinking degree of the polyamide layer is increased, the formation of the membrane with a highly hydrophilic nature is enhanced. The presence of the polyamide layer on the surface of the membrane leads to the creating of a TFC surface with more negatively charged. The negative charge of the membrane surface was decreased when the reaction time of TMC increased. The formation of −COOH groups is more in short reaction time of TMC. Since the time of TMC reaction is one of the effective parameters in crosslinking degree of interfacial polymerization; hence, it influenced the salt rejection and water flux. The formation of the amide group is a vital sign for the crosslinking degree of monomer, the hydrolyzing of acyl chloride, and film formation and growth. During the continuous reaction of TMC and PIP, it is expected that the amount of −C=O groups increased in the membranes. The presence of the more −C=O group indicated more reaction between the monomers. The existence of the −OH group is a sign of the hydrolysis of TMC. At the beginning of the reaction, the water diffusion into the membrane matrix facilitates the hydrolysis of TMC. The hydrolysis of the membrane by the aqueous phase at the initial stage (or shorter TMC reaction time) is owing to the loose structure of the coating layer. As the reaction time increased, the −OH and −C=O would be enhanced, and bonding between the OH and C–O groups would reduce. Therefore, a membrane with a dense layer would be created [51,53]. 

## 3. Improvement of Interfacial Bonding

Generally, the tubular braids and separation layers are incompatible. The thermodynamical incompatibility of these two layer may cause an apparent change in interface structure between the supported matrix and separation layer. Therefore, the interfacial bonding strength between the braid and the separation layer is a crucial issue in the braid-reinforced hollow fiber membrane. The peeling of two layers during the membrane operation process reduces its lifetime and restricts its application. Hence, the affinity (compatibility) between two layers plays a vital role in the strength of interfacial bonding. The high interfacial bonding strength is favorable in high-pressure hydraulic cleaning. The infiltration property of BHFMs is investigated by the contact angle between the braid and polymer solution [44,45,54].

### 3.1. Hybrid Braid Hollow Fiber Membranes

The selection of material for polymer and tubular braid affects the interfacial bonding performance. Relative to heterogeneous BHFM (braids and separation layer are made from different materials), homogeneous BHFM that contained the same materials in the tubular braid and the separation layer has desirable interfacial bonding strength. The interfacial bonding of the homogenous and heterogeneous membrane differed from each other. The interfacial bonding between the braid and the separation layer is poor in heterogeneous BHFM due to incompatibility, whereas the separation layer is strongly bonded with the braid in homogenous BHFM. When the heterogeneous BHFMs are subjected to pressing or stretching effect, the deformation rate will be different between the braid layer and the separation layer. Therefore, the interface of layers would be hurt through the interlaminar shear between the braid layer and the separation layer. Hence, the interfacial bonding of the heterogeneous BHFM is the main parameter restricting its application [6,7,21,28,36,44]. There is a physical force between tubular braids and the polymer casting solution in the preparation process of BHFM. This physical force consists of adhesive curing (when two surfaces combine by the physical or chemical interaction) and mechanical wedging that is related to the diffusion degree of the polymer solutions to the hybrid tubular braid and the surface roughness of the hybrid tubular braids. Poor infiltration between the hybrid tubular braids and the polymer solutions results in defects, and the interfacial bonding strength would be consequently reduced. Thus a desirable infiltration performance can considerably improve the interfacial bonding strength. Generally, the contact angle is used in order to characterize the infiltration ability. The smaller contact angle between the polymer solutions and tubular braid reveals the better the infiltration performance [6,7]. One of the methods to enhance interfacial bonding is utilizing the same material between the coating layer and the reinforced matrix. Fan et al. prepared a novel braid hollow fiber membrane consisting of a hybrid braid (containing cellulose acetate and polyacrylonitrile) and a separation layer. The resulted membrane provided a well interfacial bonding state and reduced the negative effect of CA fiber swelling on membrane permeability. Fan et al. also investigated the effect of braid composition and CA concentration on BHFM performance. They resulted that the best ratio of the fibers in the braid phase is 2/1(CA/PAN) by considering the membrane permeability and interfacial bonding state [21]. The infiltration of the polymer solution may reduce the pure water permeability (PWP). It was reported that PWP of the homogeneously BHFM is lower than heterogeneous BHFM because infiltrated polymers can be tightly incorporated in the porous braid and thus reduce the PWP [11]. 

**Figure 7:**
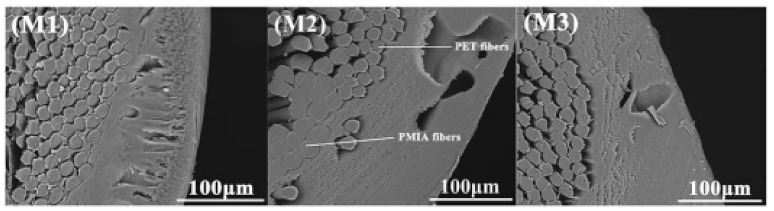
The cross-section morphologies of (**M1**) Pure PMIA for the braid (**M2**) equal composition of PMIA/PET for the braids, and (**M3**) Pure PET for the braids [28].

Zhou et al. [36] prepared a braid hollow fiber membrane with desirable antifouling properties and mechanical strength for wastewater treatment. The blending PVC with PVC-co-PEG methyl ether methacrylate) (poly(VC-co-PEGMA)) copolymer coated on PET braid. The high interfacial bonding and tensile strength indicated the good compatibility between the PET-braid and coating layer. Excellent antifouling properties, higher hydrophilicity, and BSA repulsion resulted due to the segregation of PEGMA on the membrane surface. Chen et al. [28] fabricated three BHFMs with PMIA as a polymer and different braid compositions (different ratios of PMIA/PET). As shown in Figure 7, the coating layer of pure PMIA for the braid indicated a homogeneous structure with good compatibility and firmly bonding between the reinforced braid and separation layer; whereas, poor interfacial boding and heterogeneous separation layers were observed for braid with pure PET. For the braid contained both PMIA and PET, the tightly bonded for separation layer with the PMIA fibers and the PET fibers observed; whereas, there was the poor interfacial bonding between the PET fibers.

It seems the hybrid braid is an effective method with high performance, but it is hard to apply on a large scale. It is challenging to fabricate BHFM consisting of hybrid braids (with hydrophilic polymeric) and the same material on the coating layer. Therefore, it is necessary to develop an easy, effective, and low-cost procedure to control the properties of the commercial braids [11].

### 3.2. Alkaline Pretreatment 

Alkaline pretreatment of the braid surface is a simple method for increasing and facilitating polymer adhesion. It also leads to increase braid hydrophilicity. This method provides good support for the coating layer without reducing the quality of the braid. El-Badawy et al. [4] investigated the effect of the alkaline pretreatment of the braid on BHFM morphology and performance. The membrane is treated by two alkaline solutions (KOH and NaOH). The treated membrane in KOH had the highest water flux. The investigation of the surface morphology of the braids revealed that the expansion of the braid interspaces and washing effect contribute to more porosity and permeability. 

Zhou et al. [11] prepared a BHFM by coating a blended polymer (amphiphilic copolymer/PVC) solution on a modified (alkaline-treated) PET braid. The modification by alkaline leads to endow more polar groups to PET braid and consequently more hydrophilicity. Based on the results of Zhou et al. [11], the bonding strength between the coating layer and alkaline-treated PET braid was about two times higher than the non-treated PET braid. The tensile strength of the PET braids was reduced after the alkaline treatment. The basic PET braid had more tensile strength relative to treated PET. The decreasing mechanical strength indicated that the PET braids were weakened by the hydrolysis of PET chains during the alkaline treatment. The hydrolysis process reduces the crystallinity and the molecular weight of PET. The defects created on the braids also lead to decreasing the mechanical strength. Hence, the treatment conditions (alkaline concentration, reaction time) should be optimized for obtaining a strong braid membrane. The water absorption ratio of the PET fibers increases with increasing KOH concentration and treatment time, indicating the hydrophilicity of the PET braids is improved. The increase in water adsorption is attributed to the hydrolysis of PET during the alkaline treatment when the ester groups exciting in PET are hydrolyzed to hydroxyl and carboxylate groups. Long treatment time and high alkaline concentration cause speed up the hydrolysis, binding more hydrophilic groups and improving the hydrophilicity of the braids. The highest water adsorption and the best hydrophilicity are attributed to the most porous structure of the PET braids. Therefore, the hydrophilic polymer solution can quickly infiltrate into the braid and fill the braided channel during the fabrication process. Then the braided channel is blocked during the polymer solidification in the coagulation bath. The braid with higher hydrophilicity increases the infiltration of the hydrophilic coating solutions, which reduces the porosity and pore size on the braid. Thus, PWP was significantly decreased. However, the braid’s hydrophilicity can be increased by hydrolysis, but the hydrolysis process should be optimized to avoid PWP reduction. The interfacial bonding strength between the hydrophilic PET braid and coating layer was more than the separation layer and the hydrophobic braid. After alkaline modification, carboxylates and hydroxyl groups, as the newly polar groups, result in increasing the surface energy on the braids tube. Consequently, high infiltration leads to increasing the bonding strength.

### 3.3. Modification of the Braid Surface

**Figure 8:**
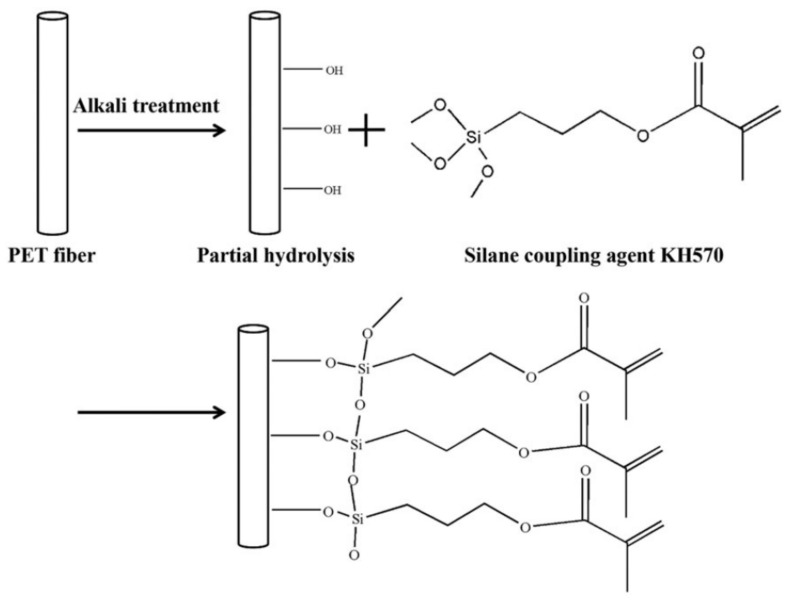
Surface modification for the braided tubes with silane coupling agent KH570 [55].

**Figure 9:**
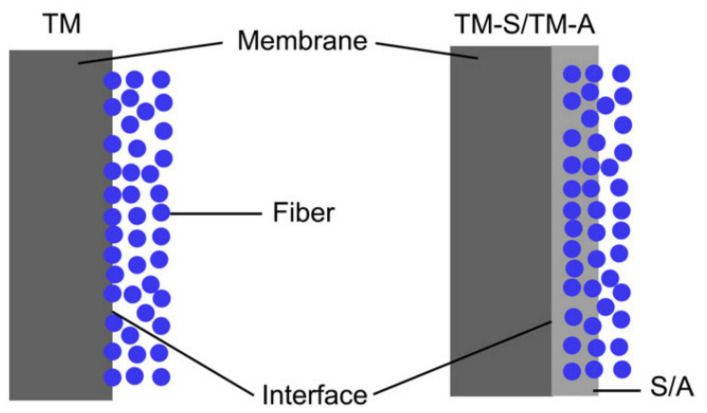
Schematic diagram of the interface [55].

Another approach for optimizing the interfacial bonding ability is the modification of the surface by coating methods. Liu et al. [55] modified the outer surfaces of the braided tubes with silane coupling agent KH570 and acrylate adhesive before fabrication of the fiber tube reinforced HFM. Based on Figure 8, coating the outer surface of the fiber tubes by acrylate adhesive and silane coupling age leads to filling the gap of the loops and the grooves between the fibers. A significant improvement in tensile strength was reported by Liu et al. in the optimum amount of modifiers dosages. When the dosage of the acrylate adhesive exceeded the optimum, the role of voids blocking was more than the adhesion on the membrane. Hence, the flux of the membrane decreased, and membrane resistance increased. It is also reported that an increase in silane coupling agent amount has a positive effect on fiber wettability and porosity due to creating spongier pores in the membrane structure. Figure 8 shows the diagram of a modification of the braid surface with the silane coupling agent. Another feasible approach was to modify the fiber tube by physical or chemical methods, such as coating with modifiers on the surface of the fiber tube or introducing chemical groups that help increase the affinity of the fiber tube to the membrane [55]. Figure 9 indicates the effect of modification of the braid surface on the interface of polymer and braid.

### 3.4. The Presence of Additive 

Peechmani et al. [24] reported that the introduction of the ZnO nanoparticles in PSf dope solution promoted the infiltration of the dope solution. It is because of the hydrophilic property of ZnO that facilitates the infiltration of the coating solution into the braided support and accumulates between the braid channels during the preparation process. More infiltration of coating solution into the braided support will enhance the mechanical stability of the BHFM due to the tight bonding between the selective layer with the braid layer that prevents the peeling of the selective layer from the braided support. ZnO nanoparticles also influenced the thickness of the separation layer. The membrane without nanoparticles had a thicker separation layer relative to the other membranes because of the lower infiltration rate and the uneven circular shape of the braid layer during the spinning process (because of mechanical stress that handled to pull the braid layer out from the spinneret).

**Table 2:** Summary of BHFM studies.

Polymer Coating Solution/wt. (%) Additive in Coating Solution/wt.(%)	Braid (Threads/Filament) Composition	Type of Spinning Methods/ Spinning Conditions	Investigated Parameters	OperationalCondition	Impurities/Application	Results	Comparison with HFM	**Ref**
**Hybrid braid**								
Polymer: CA; 10, 12, 14 (wt.%) Additive: PEG:20 wt.%	CA/PAN	Dry–wet spinning/ - coagulation bath: water (25 °C) - air-gap distance: 10 cm - take-up speed: 100 cm/min	- The effect of braid composition - The effect of polymer (CA) concentration on the structure and performance	Pressure: 0.1 MPa	BSA solution Milk solution	Max Tensile strength (MPa): 33.8 for CA14 and 62.9 MPa for pure PAN in the braid Max BSA Rejection: CA10: 90% CA12: 98% CA14: 99% Max PWF: 300 L/m^2^h for 1/2 (CA/PAN) Max bursting strength (MPa): 0.75 for pure CA in the braid Min bursting strength (MPa): 0.22 for pure PAN in the braid	NA	[21]
Polymer: PVC: 12 wt.% Additive: PVP 10 wt.%	PET/PAN	Dry–wet spinning/ - coagulation bath: water (28 °C) - air-gap distance: 12 cm Take-up speed: 66 cm/min	- The effect of braid composition on the structure and performance	Pressure: 0.1 MPa	BSA solution	Max Tensile strength (MPa): 106 for pure PETMax BSA Rejection: 70% for 1/1: PET/PAN	- Higher tensile strength relative to HFM	[6]
Polymer: PMIA 5,8,10,15 (wt.%) Additive: PVP 2 wt.%, PEG: 8, CaCl_2_:3.5, LiCl: 2.5 wt.%	PMIA/PET	Dry–wet spinning/ - Coagulation bath: water (25 °C) - Air-gap distance: 15 cm Take-up speed: 50 cm/min	- The effects of polymer concentration - The effects of braid composition	Pressure: 0.1 MPa	skim milk solution	Max PWF: 296.85 L/m^2^h for PMIA5 Max BSA Rejection: 97.9% for PMIA15 Max Tensile strength (MPa): 179.15 for PMIA15Max bursting strength (MPa): 0.98 PMIA15	NA	[28]
Polymer: PVDF: 18 wt.% Additive: PEG: 3 wt.%	PVDF- PET	NIPS: Dry–wet spinning/ - Coagulation Bath: water (25 °C) - Air-gap distance: 10 cm Take-up speed: 2 RPM	- The effect of pretreatment step by alkaline method on performance	Pressure: 0.1 MPa	NA	Max PWF: 1388 L/m^2^h for membrane treat by KOH Max Tensile strength (MPa):113 for membrane treat by KOH	NA	[4]
Polymer: PVC 6,8,10,12,14 (wt.%)Additive: PEG 5		Dry–wet spinning/ - Coagulation bath: water (20 °C) - Air-gap distance: 8 cm Take-up speed: 220 cm/min Dope solution temperature: 70 °C	- The effect of polymer Concentration - The effect of additive molecular weight		NA	Max PWF: 10.1 L/m^2^h for PVC10 Max BSA Rejection: 76.12% for PVC10 and PEG 6000	- Higher rejection Higher flux recovery rate	[29]
**Pure braid**								
Polymer: CA 6,8,10,12,14 (wt.%) Additive: PEG6000:6PEG400:10	CA	Dry–wet spinning/ - Coagulation bath: water (20 °C) - Air-gap distance: 10 cm Take-up speed: 66 cm/min Dope solution temperature: 70 °C	- The effects of polymer concentration	Pressure: 0.1 MPa	NA	Max Tensile strength (MPa): 14.2 for CA14 Max bursting strength (MPa): 0.51 for CA14 Max PWF: 220 L/m^2^h for CA6 Max BSA Rejection: 90% for CA14	NA	[37]
Polymer: PAN 8,10,12,14,16 (wt.%) Additive: PVP (7 wt.%) and Tw-80 (2 wt.%)	PAN	Dry–wet spinning/ - coagulation bath: water (25 °C) - air-gap distance: 15 cm Take-up speed: 20 cm/min Dope solution temperature: 70 °C	- The effect of polymer (PAN) concentration in coating solution - The effect of two type of braid composition	Pressure: 0.1 MPa	BSA solution	Tensile strength (MPa): 86.3 Max PWF: 345 L/m^2^h for PAN10 Max BSA Rejection: 91% for PAN18	NA	[7]
Polymer: PAN 8,10,12,14,16 (wt.%) Additive: PVP (7 wt.%) and Tw-80 (2 wt.%)	PET	Dry–wet spinning/ - coagulation bath: water (25 °C) - air-gap distance: 15 cm Take-up speed: 20 cm/min Dope solution temperature: 70 °C	- The effect of polymer (PAN) concentration in the coating solution - The effect of two types of braid composition	Pressure: 0.1 MPa	BSA solution	Tensile strength (MPa): 188 Max PWF: 470 L/m^2^h for PAN10 Max BSA Rejection: 91% for PAN18	NA	[7]
Polymer: PVC Additive: poly(VC-co-PEGMA)	PET	Dry–wet spinning/ - coagulation bath: water (24 °C) - Air-gap distance: 0.5 cm Take-up speed: 500 cm/min Dope solution temperature: 45 °C			UF BHFM for wastewater treatment		- Higher tensile strength - Higher bursting strength - Lower thickness for the coating layer	[36]
Polymer: PSf: 16 wt.% Additive: ZnO 0,0.5, 1,1.5 (wt.%)		Dry–wet spinning/ - coagulation bath: water (25 °C) - Air-gap distance: 10 cm - Take-up speed: 200 cm/min		Pressure: 0.1 MPa	1000 ppm BSA solution	Max PWF: 920 L/m^2^h for ZnO:1.5 Max BSA Rejection: 96.5% for ZnO:1.5	- Higher water flux - higher rejection	[24]
Polymer: PVDF 15,20,25,30 (wt.%) Additive: PVP20 wt.% PVDF	PET	Dry–wet spinning/	- Effect of polymer concentration in dope solution - Numbers of PET threads(n)	Pressure: 0.1 MPa	Pure water	Max tensile strength (MPa): 11.15 for PVDF20 and 3 PET threads Max PWF: 160 L/m^2^h for PVDF18 Max bursting strength (MPa): 0.45 PVDF30	- Higher tensile strength - similar separation properties	[46]
Polymer: PVDF 13 (wt.%) Additive: Ge 0,0.1,0.3,0.5,0.7 (wt.%) SiO_2_:4, DOP:10	PET	Dry–wet spinning/ - coagulation bath: water (30 °C) - Air-gap distance: 20 cm Take-up speed: 120 cm/min Dope solution temperature: 70 °C	- The effect of additive concentration	0.1 MPa	kerosene and water mixture (1:1, *v*/*v*)/oil/water separation	Max Rejection: 99.7% for Ge:0.5 PWF: 65 L/m^2^h for GE:0.5	NA	[38]
Polymer: PU 16 wt.% Additive: Ge 0.0.1,0.3,0.5 (wt.%) SA:4, NaCl:0.2	PET	Electrospinning method/ - Positive pressure of the spinneret: 25.5 kV - Negative pressure of the receiving device: 5.5 kV - Receiving distance: 10 cm - Receiving device speed: 1500 rpm - Spinning solution injection speed: 2.1 mL/h - Spinning temperature: 25 °C - Relative humidity: 5%	- The effect of additive concentration	0.1 MPa	kerosene and water mixture (1:1, *v*/*v*)/oil/water separation	Max Rejection: 99% for Ge:0.3 PWF: 1443 L/m^2^h for GE:0.3	NA	[43]
Polymer: PVDF 8,10,12,14,16 (wt.%) Additive: PVP:8 (wt.%) Tw-80:2 (wt.%)	PAN	Dry–wet spinning/ - coagulation bath: water (25 °C) - Air-gap distance: 15 cm Take-up speed: 15 cm/min Dope solution temperature: 70 °C	The effect of polymer concentration in coating solutions	Pressure: 0.1 MPa	1 g/L BSA	Max tensile strength (MPa): 75 Max PWF: 550 L/m^2^h for PVDF8 Max BSA Rejection: 95% for PVDF16	NA	[40]
Polymer: PVDF 6,8,10,14,18 (wt.%) Additive: PVP:7 (wt.%) Tw-80:3 (wt.%)	PVDF	Dry–wet spinning/ - coagulation bath: water (20 °C) - Air-gap distance: 10 cm Take-up speed: 15 cm/minDope solution temperature: 60 °C	- The effect of polymer concentration in coating solutions	Pressure: 0.1 MPa	2 g/L Egg albumen	Max tensile strength (MPa):11 for PVDF10 Max PWF: 900 L/m^2^h for PVDF6 Max BSA Rejection: 81% for PVDF18	- Higher tensile strength - Higher BSA rejection	[45]
Polymer: CA Additive: Ge	-	-	- The effect of Ge concentration in coating solutions	-	-	Max tensile strength (MPa):30 Max PWF: 158.1 L/m^2^h for Ge:1%	NA	[56]
Polymer: PA PIP: 2.0% *w*/*v* TMC: 0.13% *v*/*v*	Polymer: PSf:16 (wt.%) Additive: PVP 10 (wt.%)	Interfacial polymerization of PIP and TMC on UF support membrane	- The effect of TMC reaction time	Pressure: 0.6 MPa	MgSO4 NaCl TOC/ TFC NF	Max PWF: 5.1 L/m^2^h MgSO_4_ Rejection: 65% NaCl Rejection: 26% TOC removal:65%	NA	[51]
Polymer: PA PIP: 1.0% *w*/*w* TMC:0.1, 0.15, 0.2% *w*/*v*	PVDF and polyester	Interfacial polymerization of PIP and TMC on UF support membrane	- The effect of monomer concentration	Pressure: 0.1 MPa	MgSO4 NaCl/ TFC NF	Max PWF: 22 L/m^2^h Max MgSO_4_ Rejection: 92% NaCl Rejection: ˂30%	NA	[10]
Polymer: PVDF	Fiberglass material	-	- Estimation of the tensile strength of MF and UF hollow fiber braid membrane	-	-	Max tensile strength (MPa):10 for membrane with 0.355 Thickness	NA	[57]

PMIA: Poly (m-phenylene isophthalamide); PVDF: Polyvinylidene fluoride; poly(VC-co-PEGMA): amphiphilic copolymer poly (vinyl chloride-co-poly (ethylene glycol) methyl ether methacrylate); NIPS: non-solvent-induced phase inversion; PA: polyamide; PET: Polyethylene terephthalate (PET); Ge: Graphene; DOP: Dioctyl phthalate; PU: polyurethane; SA: Stearic acid; BSA: bovine serum albumin; PIP: piperazine; TMC: trimesoyl chloride; PWF: pure water flux; PVP; Polyvinylpyrrolidone; NA: not applicable.

## 4. Operation Parameters

An increase in operating pressure leads to the enhancement of PWF. This increase is slowed in higher operation pressure that is clear in a lower concentration of polymer. It is probably due to more compression of the porous membrane in high pressure and more resistance to the compaction for less porous membrane [46]. Peechmani et al. [24] indicated the BSA rejection was approximately constant, and the flux increased when the operating pressure increased. Xia et al. [10] tested the performance of TFC-BHFM under pressure until failure in terms of MgSO_4_ rejection and water flux. A slight enhancing of MgSO4 rejection and a linear increase was observed for water flux by the enhancement of pressure up to 0.5 MPa. The drop in salt rejection and a sharp increase in water flux up to 0.5 MPa indicates membrane delamination or tearing of the selective layer. 

**Figure 10:**
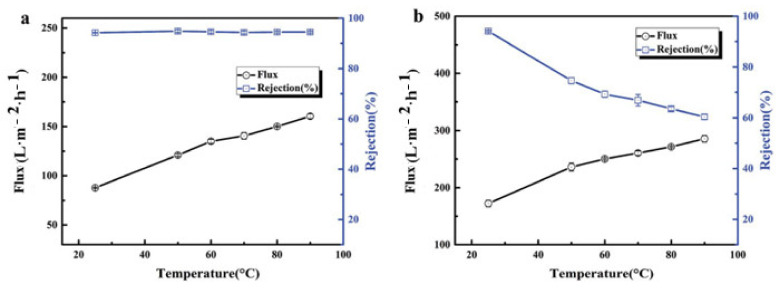
The effect of temperature on rejection and flux of (**a**) PMIA-BHFM and (**b**) PVDF-BHFM.

Chen et al. [28] reported that the increase in the operating temperature from 25 °C to 90 °C leads to an increase in water flux because of a reduction in water solution viscosity. They investigated the performance of tailoring-PMIA-BHFM and commercial-PVDF- BHFM at different temperatures. According to Figure 10, the ink solution rejection was stable for PMIA braid hollow fiber membranes, whereas it was decreased dramatically for the PVDF-BHFM. The PMIA braid hollow fiber membranes exhibited thermal stability, while the PVDF membrane faced a crucial cracking. It is the main reason for the reduction in the rejection of the ink solution. The presence of intermolecular hydrogen bonding and large benzene rings in macromolecular chains of PMIA leads to a high glass transition temperature (more than 270 °C). Hence, the pore structure is maintained, and deformation has not happened in different thermal conditions. The PMIA braid hollow fiber membranes are thus introduced for application in separation processes with high temperatures. 

## 5. Applications

Hollow fiber membranes are desirable for numerous membrane applications. They are favored for use in water treatment processes due to the desirable properties mentioned above [9]. Submerged membrane bioreactor is usually utilized to remove common pollutants from municipal and industrial wastewater because of many advantages such as high-quality effluent, low sludge production, and reduced footprint. The module can be prepared by the flat sheet or hollow fiber membranes, but the HFMs have been more widely used due to easy assembling and high permeability per installation area. However, HFM in the submerged MBR is required high mechanical properties because of easily broken during the high-pressure back-washing and cleaning process or aerated airflow [37,40,58]. Fan et al. [37] investigated the BHFM in the MBR process. The tensile strength of prepared BHFMs was higher than 11 MPa, which this content increased with the increase in polymer concentration in dope solution. 

**Figure 11:**
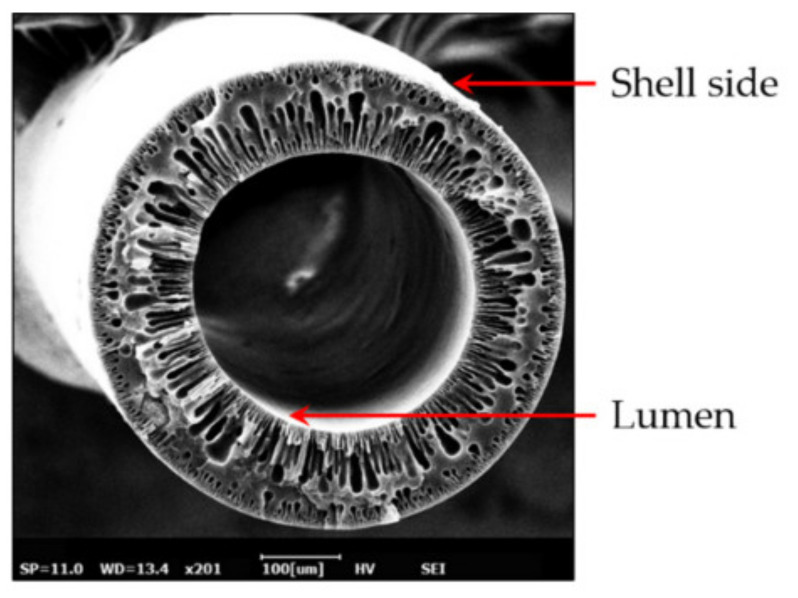
The illustration of shell and lumen side of an HFM [61].

Generally, the hollow fiber membranes are not applicable in high-pressure applications such as NF and RO processes because of their poor mechanical strength. Whereas they are good candidates for wastewater treatment, removal of heavy metals, and drinking water purification [10,15,51]. Thin-film composite (TFC) membranes are excellent candidates for RO and NF processes in water and wastewater treatment applications due to the ultrathin selective layer. The TFC design possesses desired performance in terms of salt rejection and permeability when united with asymmetric membranes. The cross-linked aromatic polyamide as a selective layer on the ultrafiltration membrane porous or other supporting substrates fabricated by interfacial polymerization of proper monomer (e.g., trimesoyl chloride (TMC)) is the most successful and famous commercial product in the last decades [10,50,59]. As mentioned, TFC membranes consist of a support layer and selective layer. The selective layer that typically cross-linked polyamide is a thin, dense, and poor mechanical film with high selectivity. The second layer is a porous film that plays the role of the substrate with high mechanical strength under pressure. This layer generally is formed from polyethersulfone and polysulfone. Figure 11 illustrates the shell and lumen side of HFM. Generally, it is difficult to apply a thin film on the shell or lumen side of the HFM during fiber manufacturing. The HFMs designed for liquid filtration have large lumens due to reducing resistance in mass transfer and pressure drop liquid stream. In high-pressure applications, the walls of HFMs must be thicker, but it is not desirable for liquid flow applications. It is reported several studies on the fabrication of HFM with high-pressure tolerance and improvement of mechanical stability. These studies propose using a polymer/additive with intrinsic high mechanical strength, optimization of spinning parameters, and braid reinforced composition [10,60]. Xia et al. [10] fabricated a TFC-HFM that uses a braid-reinforced ultrafiltration-HFMs as a substrate, consisting of a PVDF coating layer and polyester braid. The selective layer is formed using interfacial polymerization of TMC and piperazine (PIP). The presence of the reinforced support devoted high-pressure endurance proprieties to the HFM. The prepared membranes could tolerate pressure up to 0.5 MPa while possessing high selectivity for divalent/monovalent ions. Turken et al. [51] fabricated reinforced TFC-HF NF based on PSf ultrafiltration membranes and polyamide layer prepared from PP and TMC monomers. The membranes were investigated in solutions with different organic matter and salts. Their results evidenced the prepared membranes are a good candidate for water-treatment applications.

Another application of BHFMs is the oil/water separation process. Oily wastewater is produced by different industries such as metal finishing industries, leather, food, petrochemical, oil exploration, refining, and transportation of oil products. This type of wastewater is a severe threat to human health and the environment. Membrane technology is a proper option for oily wastewaters treatment owing to advantages such as low costs, no secondary pollution, high energy efficiency, sustainability, and no additives. Two parameters are essential for the oil/water separation process: the selective wettability for oil or water (lipophilicity/hydrophobicity) that provide the required driving force and the connected pores with a suitable pore size for filtration. Both hydrophobic and hydrophilic membranes could be utilized for the separation of oil/water. Hydrophilic membranes increase the water permeation relative to oil permeation leading to higher water flux and good antifouling properties. Nevertheless, the hydrophilic membranes must provide a large volume of water permeate fluxes in separations of oil/water. They required high energy consumption and large membrane areas. Generally, the present pollutants in oily wastewater are mainly due to the oil phase, but their contents are relatively low. Therefore, hydrophobic membranes are better candidates for oily wastewater treatment based on workload [38,43]. Hao et al. [38] prepared (PET-) braid-reinforced PVDF/graphene hollow fiber membranes. PVDF-BHFM shows appropriate hydrophobic properties for oil/water separation and good mechanical strength. Graphene was also used to enhance the hydrophobicity of the membrane. Their results showed that the prepared hydrophobic BHFMs ultimately rejected water during the separation process.

## 6. Conclusions

Hollow fiber membranes (HFMs) are a good candidate for the membrane separation process due to desirable properties such as high permeability and surface area, good filtration efficiency, small footprint, etc. However, they are often possible to break during the high-pressure cleaning and aeration process. Tubular braids a supported is proposed to improve the mechanical strength of HFMs due to high tensile strength. The peeling of the surface layer from the tubular braid is the drawback of the BHFM due to thermodynamic incompatibility. Depending on the type of application, the kind of polymer/additive and their content are the essential parameters that affect the performance of BHFMs. PAN, PVC, CA, PSf, and PVDF are the common polymers used in BHFM preparation. The interfacial bonding strength between the braid and the separation layer is an essential issue in BHFMs. Because the separation of two layers reduces its lifetime and limits its application; hence, the affinity between the two layers will be improved. The interfacial bonding strength between the braid and the separation layer is an essential issue in BHFMs. Hence, the affinity between the two layers will be improved by hybrid braids, alkaline pretreatment, and the use of additives. Recently, the BHFMs have been used in RO and NF applications. Although it is required a comprehensive investigation that optimizes the membrane performance in terms of flux, mechanical properties, rejection, and fouling; it is expected that the applications of these types of the membrane will be enhanced in different aspects and the gap between studies and apply in large scales would be reduced.

## Figures and Tables

**Table 1:** Used-polymers for BHFM and their properties.

**Polymer**	Chemical Structure.	Advantages	Disadvantages/Improvement Approach	Application	Ref.
**PAN**	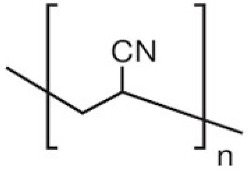	- Low price - Excellent aging-resistance - High hydrophilicity - Good stability - Good solvent resistance	- Low mechanical stability	Water, municipal, and industrial wastewater treatment	[7,31]
**PVC**	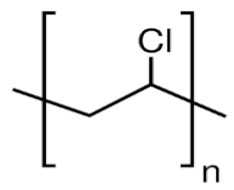	- Excellent mechanical strength - High corrosion resistance - Low cost	- Hydrophobic nature/ Using amphiphilic copolymer	Ultrafiltration BHFM for wastewater treatment	[36]
**PSf**	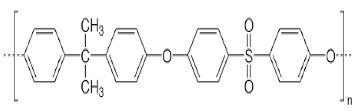	- Excellent mechanical strength - Stability at pH levels from 2 to 13 - Excellent resistance to caustic - Good resistance to moderate chlorine - Operating at high temperature and pressure	- Hydrophobic nature/ Incorporation of zinc oxide (ZnO)	Wastewater treatment	[24]
**CA**	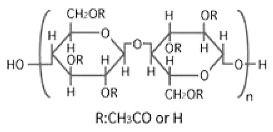	- Good film forming - Relatively low cost - High flux - Good toughness - Biocompatibility	- Poor mechanical property/Using homogeneous braid reinforced	Wastewater treatment	[21,37]
**PVDF**	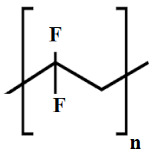	- Good hydrophobic property [38] - Semi-crystalline polymer - Good mechanical strength - Stability against vigorous chemicals - Good thermal stability	- dramatically decreasing of hydrophobicity in continuous use/ blending method for improving the hydrophobicity (Graphene) [38] - hydrophobic property [30]	oil/water separation [38] wastewater treatment [30]	[38,39,40,41,42]
**PMIA**	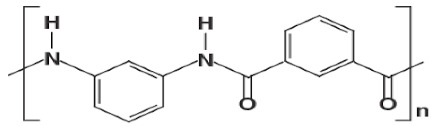	- High thermal stability - Excellent mechanical properties - Hydrophilic property	NA	wastewater treatment	[28]
**PU**	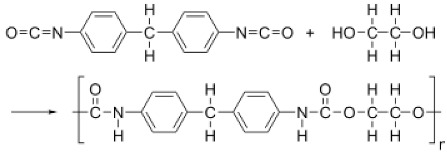	- Biodegradability - Biocompatibility - Low cost	NA	oil/water separation	[43]

PAN: Polyacrylonitrile; PVC: Polyvinylchloride; PSf: Polysoulfone; CA: Cellulose acetate; PVDF: Polyvinylidene fluoride; PMIA: Poly (m-phenylene isophthalamide); Pu: polyurethane; NA: not applicable.

## Data Availability

Not applicable.

## Abbreviations

HFM:	Hollow fiber membrane
BHFM:	Braid Hollow fiber membrane
PMIA:	Poly(m-phenylene isophthalamide)
PVDF:	Polyvinylidene fluoride
Poly(VC-co PEGMA):	Poly(vinyl chloride-co-poly(ethylene glycol) methyl ether methacrylate)
NIPS:	Non-solvent-induced phase inversion
PA:	Polyamide
PET:	Polyethylene terephthalate
Ge:	Graphene
DOP:	Dioctyl phthalate
PU:	Polyurethane
SA:	Stearic acid
BSA:	Bovine serum albumin
PIP:	Piperazine
TMC:	Trimesoyl chloride
PWF:	Pure water flux
